# Ether and Chloroform

**Published:** 1853-09

**Authors:** 


*Ether and Chloroform.* M. Jobert de Lamballe thus compares these anesthetics—

Ether has a strong, penetrating odor, which renders it repugnant to some persons; it always commences by irritating the respiratory organs, it frequently provokes coughing, and sometimes suffocation. Chloroform does not disturb nor irritate the organs it passes through; on the contrary, patients take pleasure in inhaling it. Chloroform produces only a feeble organic excitement; ether would seem to cause a violent one, as the inspirations of the latter give rise to a good deal of agitation in the heart and in the other muscles. Ether develops its anesthetic effects slowly, and they remain for some time after the experiment or the operation is ended, in the form of intoxication, headache, feeble pulse, and cold limbs. Chloroform, on the contrary, ceases, in general, its action when the patient stops inhaling it, and it is only in especial cases it is seen to prolong its effects for some time after the patient ceases to inhale it—cases which never occur except where the operator has pushed the saturation of the system to an extreme degree. Ether alters the color and the consistency of the blood, while chloroform does not change either its nature or its color. Chloroform never retards the healing of the wounds, ether frequently militates against their healing. M. Jobert de Lamballe has never observed chloroform diminish in any way the productions engendered by, and necessary to, the healing process, or alter their consistency; ether, on the contrary, seems to produce marked effects, making the plastic lymph less consistent and less vital. Both chloroform and ether excite at first the vascular apparatus, precipitating the motions of the heart as if the latter was disturbed by the introduction of some foreign body. Ether produces these effects in a much greater degree than chloroform, and continues them almost indefinitely, i e., during nearly the whole time of the experiment, or the operation. Ether acting upon the organs it touches, under the form of vapor, has a tendency to inflame them; chloroform produces nothing of the sort. Chloroform and ether, in their second action, stupefy the nervous system, and consequently suspend the functions of the muscles of locomotion and of organic life. Chloroform paralyzes them hopelessly, as in a second the heart may cease to beat. Chloroform produces its effect instantly; in thirty seconds, in a minute and a half, in two, three, or four minutes at most. Ether, on the contrary, determines insensibility only in 13, 15, 18, or 20 minutes, and sometimes requires even more time. Chloroform calms the organs; ether troubles them in a violent manner, even during sleep, which is accompanied with agreeable or painful dreams. Ether excites, especially women, in a way which forbids its being administered publicly; while chloroform makes all persons gay, a gaiety which may be indulged in before witnesses of both sexes. The sequent accidents produced by ether are phenomena of inflammation; those following chloroform are symptoms of feebleness and of organic weakness. Ether produces death, during the experiment, very rarely, and with great difficulty, while chloroform may determine life instantly, when the patient is not watched, or the chloroform awkwardly administered, or when the bronchia have a large communication with the sanguineous pulmonary organs, and when the chloroform is absorbed in the form of abundant vapors. In no case should chloroform be resorted to when a grave disturbance of the functions exists, dependent upon a profound lesion of the central organs of circulation or nervous swellings. It may easily be understood that a new disturbance of the functions being added to that existing, should produce a rapid and, so to say, instantaneous death. Chloroform, consequently, should never be used when the nervous system has been enfeebled by a violent shock—a gun-shot wound —or when the patient is exhausted by a long and an abundant suppuration, by losses of blood, or a chloratic state carried to an advanced degree. When chloroform has suspended the vital forces, and death is apparent, the surgeon must not abandon the patient until he has endeavored *for a long time* to recall him to life. The skin should be partially excited by cold water, frictions with alcoalats, alcali, etc., etc., the organs should be reänimated by currents of air directed upon the face and the limbs, while the breast is agitated by slight communicated motions; care must be taken to place the patient in the position most favorable to the reëstablishment of the circulation by putting him horizontally upon his back, or obliquely upon his side. Excitants introduced in his mouth; mint water; antispasmodics placed upon the rectal surface, favor the resuscitation of the motions of the heart, reduced to oscillation or complete resolution; cauterizations in the mouth or in the pharynx with ammonia, may contribute to restore the dying man; there are also examples of electricity being successfully applied to vivify an organization about stagnating forever.

